# Efficacy of the Bushen Jianpi Huoxue Formula on Beclin-1/Bcl-2-mediated autophagy and apoptosis in osteoblasts

**DOI:** 10.3389/fphar.2024.1513298

**Published:** 2025-01-06

**Authors:** Yanping Lin, Rui Zhao, Jiachun Huang, Tongying Chen, Haolin Yang, Haiwei Guo, Lei Wan, Zhihai Zhang, Ying Li, Genfu Zhu, Hongxing Huang

**Affiliations:** ^1^ Department of Orthopedics, The Third Affiliated Hospital of Guangzhou University of Chinese Medicine, Guangzhou, China; ^2^ The Third Clinical Medical School, Guangzhou University of Chinese Medicine, Guangzhou, China; ^3^ Department of Spine and Orthopaedics, The Third Affiliated Hospital of Guangzhou University of Chinese Medicine, Guangzhou, China; ^4^ Department of Osteoporosis, The Third Affiliated Hospital of Guangzhou University of Chinese Medicine, Guangzhou, China

**Keywords:** osteoporosis, osteoblasts, autophagy, apoptosis, Beclin-1, endoplasmic reticulum stress, traditional Chinese medicine

## Abstract

**Background:**

The Beclin-1/Bcl-2 complex plays a pivotal role in regulating both autophagy and apoptosis in osteoblasts affected by osteoporosis. This study first investigates whether the Bushen Jianpi Huoxue Formula can enhance the cellular function of osteoblasts. Additionally, it initially explores the functional mechanism of Beclin-1/Bcl-2-related apoptosis.

**Methods:**

Osteoblasts were isolated from the calvaria of three-day-old Sprague-Dawley female rats. The lyophilized power of the Bushen Jianpi Huoxue Formula was prepared from the following ingredients: Fructus Psoraleae, Epimedii Folium, Desertliving Cistanche, Prepared Rehmannia Radix, Radix paeoniae alba, Astragali Radix, Semen Cuscuta, Radix Salviae miltiorrhizae, Angelica sinensis, and Jujube. The primary components were detected by HPLC-MS. Beclin-1 overexpressed osteoblasts were constructed by transfection. Gene expression and protein level were examined by qRT-PCR and immunoblotting assay. Cell viability, apoptosis, and autophagy were assayed with CCK-8, flow cytometer, MDC staining, and Lyso-Tracker staining, respectively. Osteogenic differentiation was assayed by ALP staining, and mineralization by ARS staining. The complex of Beclin-1/Bcl-2 was detected by immunoprecipitation.

**Results:**

The results of this study indicated that the Bushen Jianpi Huoxue Formula could enhance both the proliferative activity, differentiation and mineralization of osteoblasts induced by Beclin-1 overexpression. This may be related to its role in activation of the WNT/β-CATENIN by increasing protein expression of WNT1 and β-CATENIN more than 1-fold. The formula effectively inhibited autophagy rate and apoptosis rate of osteoblasts by 50%. Furthermore, the formula was effective in attenuating endoplasmic reticulum stress by decreasing protein expression of AFT4, CHOP, eIF2α, and GRP78 more than 50%, which may play functions by suppressing the PERK signaling pathway. However, Mif treatment significantly weakened the effects of the formula.

**Conclusion:**

Bushen Jianpi Huoxue Formula effectively enhanced the osteogenic activity by inhibiting Beclin-1-induced autophagy instead of the binding of Beclin-1 and Bcl-2. This underscores the formula’s multifaceted role in promoting bone health and managing cellular stress, and offers novel insights into its therapeutic potential against osteoporosis.

## Introduction

Osteoporosis is a metabolic bone disorder characterized by reduced bone mass and structural deterioration, resulting in increased bone fragility and greater susceptibility to fracture (Consensus development conference, 1993). As the global aging trend intensifies, osteoporosis is increasingly emerging as a dreaded “invisible killer” that seriously threatens the lives and health of the middle-aged and elderly population (Tran et al., 2021). Epidemiologic data showed that the prevalence of osteoporosis in China varies, with rates of 4.6% in men and 16.4% in women aged 50–59 years, 5.4% in men and 37.1% in women aged 60–69 years, and 12.3% in men and 67.5% in women aged 70–79 years (Boya et al., 2013). The commonly used medications for the treatment of osteoporosis in Western medicine include bisphosphonates, hormone replacement therapy, calcitonin, parathyroid hormone analogues, and denosumab. However, long-term use of these treatments can lead to side effects such as gastrointestinal issues, osteonecrosis of the jaw, atypical femur fractures, and cardiovascular diseases, among others. Traditional Chinese Medicine (TCM) has gained popularity due to its remarkable effectiveness in treating various orthopedic conditions, including osteoporosis.

TCM categorizes osteoporosis under the category of “bone impotence,” and its primary pathological factors are “kidney deficiency,” “spleen deficiency,” and “blood stasis.” Consequently, Chinese medicine adopts the principles of “nourishing the kidney,” “fortifying the spleen,” and “enhancing blood circulation” as foundational approaches to the diagnosis and management of osteoporosis (Hong-Xing et al., 2019). The Bushen Jianpi Huoxue Formula comprises 10 traditional Chinese medicines, including Fructus Psoraleae, Epimedii Folium, Desertliving Cistanche, Prepared Rehmannia Radix, Radix paeoniae alba, Astragali Radix, Semen Cuscuta, Radix Salviae miltiorrhizae, Angelica sinensis, and Jujube. This formula is designed to tonify the kidneys, activate the blood, strengthen the spleen, benefit qi, improve blood circulation and eliminate blood stasis. However, the molecular mechanisms by which this formula acts on osteoporosis have not yet been fully clarified.

Apoptosis and autophagy are two strictly regulated biological processes in the cell that play a key role in maintaining tissue homeostasis and development (Titorencu et al., 2014; Levine and Kroemer, 2019). Dysregulation of autophagy and apoptosis under stress conditions have been strongly implicated in the development of osteoporosis (Levine and Kroemer, 2019; Parzych and Klionsky, 2014; Ma et al., 2021; Zou et al., 2021). Autophagy-related gene, Beclin-1, affects osteogenic mineralization, the differentiation of pre-osteoblasts and the transition of osteoblasts to osteoclasts (Yin et al., 2019; Chiang et al., 2018; Fernández et al., 2018). Bcl-2, a pivotal member of the Bcl-2 protein family, possesses all four Bcl-2 Homology (BH) domains that play a crucial role in inhibiting apoptosis in osteoporosis (Ma et al., 2021; Zou et al., 2021; Kale et al., 2018). Disruption of the BH3-only structural domain in Beclin-1 or the BH3 receptor structural domain in Bcl-2 leads to the formation of the Beclin-1/Bcl-2 complex, which has deleterious effects. On one hand, Beclin-1 loses its ability to counteract the anti-apoptotic function of Bcl-2, and on the other hand, Bcl-2 diminishes the autophagy-promoting activity of Beclin-1. This disruption severely disrupts the metabolic homeostasis of cells and becomes an important pathogenic factor for cellular senescence and death (Kang et al., 2011). Therefore, reducing the Beclin-1/Bcl-2 complex ratio in osteoblasts enhances autophagy and reduces apoptosis, which is a potential new option for the treatment of osteoporosis.

Previous research has demonstrated that the formula can elevate Bcl-2 protein levels in rat skeletal muscle and osteoblasts, significantly boost Bone Mineral Density (BMD), and modulate the opening extent of mitochondrial permeable transition pores in osteoporotic skeletal tissue (Ji-Li et al., 2018; Ying et al., 2013). However, the impact of Beclin-1/Bcl-2 binding on osteoblastic differentiation and mineralization remains unexamined. Then this study investigated the effects of the Bushen Jianpi Huoxue Formula on autophagy, apoptosis, differentiation, and mineralization of osteoblasts. Further Beclin-1 mutant osteoblasts were constructed to study whether the formula affects autophagy, apoptosis, differentiation and mineralization of osteoblasts through Beclin-1/Bcl-2 complex. This study may offer a new strategy for the prevention and treatment of osteoporosis, providing an experimental basis for utilizing TCM in this context.

## Materials and methods

### Formula preparation

The Bushen Jianpi Huoxue Formula consists of the following components: Fructus Psoraleae, Epimedii Folium, Desertliving Cistanche, Prepared Rehmannia Radix, Radix paeoniae alba, Astragali Radix, Semen Cuscuta, Radix Salviae miltiorrhizae, Angelica sinensis, and Jujube (Jiang-Tao et al., 2024). The detailed dosages for each component are presented in Table 1. All the ingredients were acquired from the pharmacy at the Third Affiliated Hospital of Guangzhou University of Traditional Chinese Medicine. After crushing, 100 g of the Bushen Jianpi Huoxue Formula powder was added to 1 L of pure water, boiled for 1 h. The supernatant was then extracted. Subsequently, 800 mL of pure water was added to the residue, and the suspension was boiled for 1 h. This process was repeated twice. The suspension was pooled and concentrated to 500 mL using a rotary evaporator at 60°C. The paste was then obtained, sealed in foil and stored in the refrigerator at −80°C to freeze for more than 48 h. Afterwards, the paste was subjected to freeze-drying for 72 h. The obtained lyophilized powder of the Bushen Jianpi Huoxue Formula was stored at −20°C.

**TABLE 1:** The components of the Bushen Jianpi Huoxue Formula.

Chinese name	English name	Plant source (Latin name)	Herbal part	Doses (g)^a^
Bu-gu-zhi	Fructus Psoraleae	Psoralea corylifolia Linn.	Fruit	13
Yin-yang-huo	Epimedii Folium	Epimedium brevicornu Maxim.	Leaf	16
Rou-cong-rong	Desertliving Cistanche	Cistanche deserticola Ma.	Stem	16
Shu-di-huang	Prepared Rehmannia Radix	Rehmannia glutinosa Libosch.	Tuber	16
Bai-shao	Radix paeoniae alba	Paeonia lactiflora Pallas	Root	13
Huang-qi	Astragali Radix	Astragalus membranaceus (Fisch.) Bunge	Root	16
Tu-si-zi	Semen Cuscuta	Cuscuta chinensis Lam.	Seed	16
Dan-shen	Radix Salviae miltiorrhizae	Salvia miltiorrhiza Bunge	Tuber	16
Dang-gui	Angelica sinensis	Angelica sinensis (Oliv.) Diels	Root	11
Suan-zao	Jujube	Ziziphus jujuba Mill.	Fruit	6

^a^ The formula dosage is intended for daily use by an adult human.

### High performance liquid chromatography-mass spectrometry (HPLC-MS) detection

HPLC-MS was used to detected bioactive compounds of the formula (Menezes Gama et al., 2023). The formula (100 µL) was added with 100 µL of methanol and vibrated for 10 min. The mixture was centrifuged (20,000 × g) for 10 min at 4°C. The supernatant was collected and filtered using a 0.22 µm filter membrane. The resulting sample was analyzed using an Ultimate 3000 RS system (Thermo Fisher Scientific, MA, United States), equipped with a Thermo Hypersil GOLD column (φ 2.1 × 100 mm, 1.9 μm). MS spectra were obtained using a Q executive high-resolution mass spectrometer (Thermo Fisher Scientific). The mobile phases used were (A) 0.1% formic acid in water and (B) 0.1% formic acid in acetonitrile. The gradient elution program was set as follows: 0–1 min, 2% B; 1–5 min, 2%–20% B; 5–10 min, 20%–50% B; 10–15 min, 50%–80% B; 15–20 min, 80%–95% B; 20–25 min, 95% B; and 26–30 min, returning to 2% B. Chromatographic analysis was conducted at 35°C with a flow rate of 0.3 mL/min, and the injection volume was 15 µL. The capillary temperature was set to 300°C, with argon used as the collision gas. Nitrogen served as both the sheath gas and auxiliary gas, with the auxiliary gas heater temperature set at 350°C. The analysis was performed using positive/negative ion-switching ESI mode. All measurements were performed with a scan range of m/z 100.0–1,500.0. Compound Discoverer software 3.0 (Thermo Fisher Scientific) was used to analyze HPLC-MS raw data. The substances were identified using the mzCloud database.

### Isolation of osteoblasts from neonatal rat calvaria

Three-day-old Sprague-Dawley female rats were euthanized and sterilized with 70% ethanol. The skin and brain tissue were removed from the skull using a scalpel and tweezers. The jaw was cut away, and any excess tissue and cartilage was carefully scraped off from around the calvaria. The calvaria was cut in half and placed in a flat-bottomed 5-ml tube, washed in PBS twice, incubated in 0.25% trypsin for 10 min at 37°C. Then the calvaria was incubated in 0.2% collagenase solution for 30 min at 37°C twice. The digest was transferred to a 15-mL centrifuge tube, and the cells were collected by centrifugation (1,500 g, 5 min). The obtained cells were resuspended in α-MEM medium (Gibco). The cells were incubated at 37°C in an atmosphere of 5% CO_2_ and saturated humidity. When the cells reached approximately 80% confluence, they were detached using an enzymatic digestion process and then sub-cultured at a 1:2 split ratio. The isolated osteoblasts were identified by Alkaline phosphatase (ALP) staining and Alizarin Red S (ARS) staining. All animal experiments were approved by the Committee of Ethics in Animal Research of The Third Affiliated Hospital of Guangzhou University of Chinese Medicine, Guangzhou, China.

### Preparation of drug-containing serum

After dissolution, the Bushen Jianpi Huoxue Formula was used for cellular intervention, and the concentration of Bushen Jianpi Huoxue Formula powder was selected based on the data from an *in vivo* cytotoxicity test published previously (Lei et al., 2018). The optimal dose of the formula was 4.8 g/kg. Sixteen rats were randomly divided into two groups: control group (*n* = 8) and drug group (*n* = 8). The rats in the drug group received an intragastric administration of 4.8 g/kg of the formula, while the control group was administered saline solution. The drug was given twice daily for three consecutive days. Two hours after the final gavage, blood was collected from the abdominal aorta. The collected blood was allowed to sit for 1 h, then centrifuged at 3,000 rpm for 20 min to obtain the serum. The serum was sterilized and stored at −20°C.

### Cell treatment or study design

In the control group, osteoblasts were cultivated in α-minimum essential medium supplemented with 10% (v/v) fetal bovine serum, 100 U/mL penicillin, and 100 μg/mL streptomycin at 37°C in a humidified atmosphere of 95% air and 5% CO_2_. In the Blank group, osteoblasts were maintained in medium containing 20% serum without the drug, while osteoblasts in the Drug group were cultured in medium supplemented with 20% drug-containing serum.

As a key regulator of autophagy, Beclin-1 plays an important role in differentiation and mineralization. Beclin-1 mutant (MUT) cells were constructed to study the effects of autophagy on cellular phenotypes of osteoblasts, including differentiation and mineralization. Osteoblasts in the NC group only treated with transfection reagent. Osteoblasts in the Vector group were transfected with the empty plasmid. In the MUT group, osteoblasts were transfected with plasmid carrying Beclin-1 protein-encoding sequence.

To induce autophagy of osteoblasts, Rapamycin (Rapa) (Selleck Chemicals) was used as the autophagy activator. Bafilomycin A1 (Baf) (Selleck Chemicals) served as the autophagy inhibitor. Mifepristone (Mif) (Selleck Chemicals) was utilized to inhibit the binding of Beclin-1 to Bcl-2.

### Transfection

To construct cell lines overexpressing Beclin-1, osteoblasts were transfected with plasmid carrying Beclin-1 protein-encoding sequence (MUT) using Lipofectamine 2000 (Thermo Fisher Scientific). Beclin-1 WT group received an empty plasmid (Vector). Osteoblasts in the NC group only received the reagent. The transfection was performed using the mixture of DNA and lipofectamine 2000 reagent at a ratio of 1:2. The mixture was incubated at room temperature for 20 min before transfection. Osteoblasts were incubated with the mixture at 37°C for 48 h.

### qRT-PCR

Total RNA was extracted from osteoblasts using TRIzol reagent (Takara, Dalian, China) and converted into cDNA using T4 RNA Ligase Kit (Invitrogen). Beclin-1 sequence was amplified by PCR using rTaq DNA Polymerase (Sigma) and ligated into pCDNA3.1 (Thermo Fisher Scientific). Specific primers were designed referring to the sequences of the Beclin-1 gene published in the GenBank Database of NCBI. The PCR reactions were run on fluorescence quantitative PCR instrument (ABI), with the following primers for Beclin-1: forward 5′-TCA​AGG​TCA​CTG​GAG​ACC​TT-3′ and reverse 5′-GAC​ATC​ATG​TCT​GGC​CAG​AC-3′. GAPDH served as the internal control.

### Cell counting kit-8 (CCK-8)

Osteoblasts were seeded in 96-well plates at 4,000 cells per well. Four replicate wells were assigned to each group. At approximately 50% confluence, the cells were treated with the drug. After 48 h, the drug-containing supernatant was removed. Then 100 μL of serum-free 1640 medium and 10 μL of CCK-8 reagent were added into each well and incubated for 2 h. The absorbance at 450 nm was measured using a microplate reader.

### Apoptosis assay

After treating the cells with the formula for 48 h, the contents of each well were transferred to a 15 mL centrifuge tube. The cells were washed in PBS, and then the cells were collected by centrifuged at 700 g for 5 min. The supernatant was discarded, and the cells were resuspended in PBS. This process was repeated, and the cells were then counted. A suspension of 100,000 cells was transferred to EP tubes, centrifuged at 1,000 g for 3 min. The PBS was discarded, 195 µL of buffer to each tube, and the cells were gently resuspended. Apoptotic cells were detected using the Annexin V-PE/7-AAD Apoptosis Kit (Cluster). After adding 5 µL of Annexin V-FITC and 10 µL of 7-AAD to each tube, the well was mixed using a vortex mixer and incubated for 15 min at room temperature without exposure to light. The samples were immediately analyzed using a flow cytometer.

### Monodansylcadaverine (MDC) staining

MDC staining was used as a specific marker for autophagosomes. MDC staining solution (Sigma) was dissolved in PBS. Adherent cells were incubated with MDC for 30 min at 37°C. After rinsing in washing buffer three times, MDC combined with acidic autophagic vesicles were observed using a fluorescence microscope (excitation wavelength/emission wavelength = 355 nm/512 nm).

### Lyso-Tracker staining

Lyso-Tracker staining was used to stain autolysosomes. The Lyso-Tracker working solution was prepared by dilution with DMEM medium at a ratio of 1:20,000. After treatment, the cell culture medium was replaced with the appropriate volume of Lyso-Tracker working solution, and the cells were incubated at 37°C in 5% CO_2_ for 30–120 min in the dark. After incubation, the cells were washed to remove excess staining solution, and fresh DMEM medium was added. Lysosomal staining was observed using fluorescence microscopy.

### ALP staining

To determine osteogenic differentiation, ALP activity was assayed as an early differentiation marker. Osteoblasts were inoculated into 24-well plates, and the medium was added after the cells had adhered to the walls and morphologically stabilized in culture for 24 h; thereafter, the medium was replaced once every 48 h. On the 7th day of culture, the cells were stained and tested for activity using the commercial kit (Beyotime). After aspirating the medium, the cells were rinsed twice with PBS, fixed with 4% paraformaldehyde for 15 min, the fixative was removed, rinsed once with PBS, and the alkaline phosphatase staining solution was prepared according to the instructions, and stained for 1 h. The reaction was terminated by the addition of PBS, and the cells were observed and imaged under Olympus inverted microscope (Olympus).

### ARS staining

ARS staining was performed to determine the degree of mineralization in osteoblasts cultures. Osteoblasts were plated in 24-well plates and cultured in α-MEM medium. Once reaching confluency condition, osteogenic differentiation was initiated by 50 μg/mL ascorbate and 10 mmol/L β-glycerophosphate in α-MEM medium. The medium was replaced every 48 h. On day 14, cells were washed in PBS twice, fixed in 50% ethanol for 10 min, and rehydrated in 1 mL of distilled water for 5 min. Then cells were stained using 200 µL of ARS stain (1%, pH 4.0) for 3 min at room temperature. After washing three times with distilled water and 70% ethanol, ARS-stained cultures were incubated with 100 mmol/L cetylpyridinium chloride for 1 h. Then ARS-bound calcium was released to the solution. Mineralization formation was visualized using Olympus inverted microscope (Olympus).

### Immunoblotting assay

The cells were lysed using RIPA lysing solution, and cellular protein was quantified. After electrophoretic separation using PAGE Color Rapid Gel Preparation Kit, the proteins were transferred on to the PVDF membrane. The proteins were sealed using 5% non-fat milk for 1 h, followed by incubation with primary antibodies against Bcl-2 (1:2000); Bax (1:5000); Caspase-9 (1:1000); Caspase-3 (1:2000), GAPDH (1:2000) (CST), OPG (1:5000), RUNX2 (1:2000), Beclin-1 (1:2000), ATF4 (1:1000), CHOP (1:5000), eIF2α (1:5000), GRP78 (1:5000), ALP (1:5000), OPN (1:5000), WNT (1:5000), β-CATENIN (1:1000) (Abcam) overnight at 4°C. The membranes were washed in T-TBS three times. After incubation with the secondary antibodies (1:2000; Abcam) for 1 h at room temperature, the blots reacted with Chemiluminescent Substrate. Bio-Rad Chemi Doc system was used to capture protein signal, and relative band intensities were calculated using the ImageJ software. GAPDH served as a reference protein.

### Co-immunoprecipitation

After treatment with protease inhibitors, the cells were lysed with RIPA Lysing Solution for 30 min on ice. The supernatant was centrifuged at maximum speed for 30 min at 4°C. Next, 1 μg of antibodies against Bcl-2 (1:50; ab32124; Abcam) and Beclin-1 (1:30; ab207612; Abcam) was added to the lysate and incubated overnight. Protein A/G Sepharoses (Share-bio, Shanghai, China) were then added to the cell lysate and the mixture was incubated for 4 h at 4°C with gentle agitation. Then, 10 μL of target protein-bound agarose beads were collected, washed three times with PBS, and centrifuged at 3,000 rpm for 3 min each time. The beads were then resuspended in 2 × SDS-PAGE loading buffer, and boiled for 5 min. The immunoprecipitates were then subjected to immunoblotting assay.

### Statistical analysis

Data were analyzed using GraphPad Prism 9 and SPSS 26.0. Continuous variables are presented as means ± standard deviation (mean ± SD). One-way ANOVA was employed for comparisons involving two or more groups. A p-value of less than 0.05 indicates significant differences between groups.

## Results

### Identification of bioactive compounds

**FIGURE 1:**
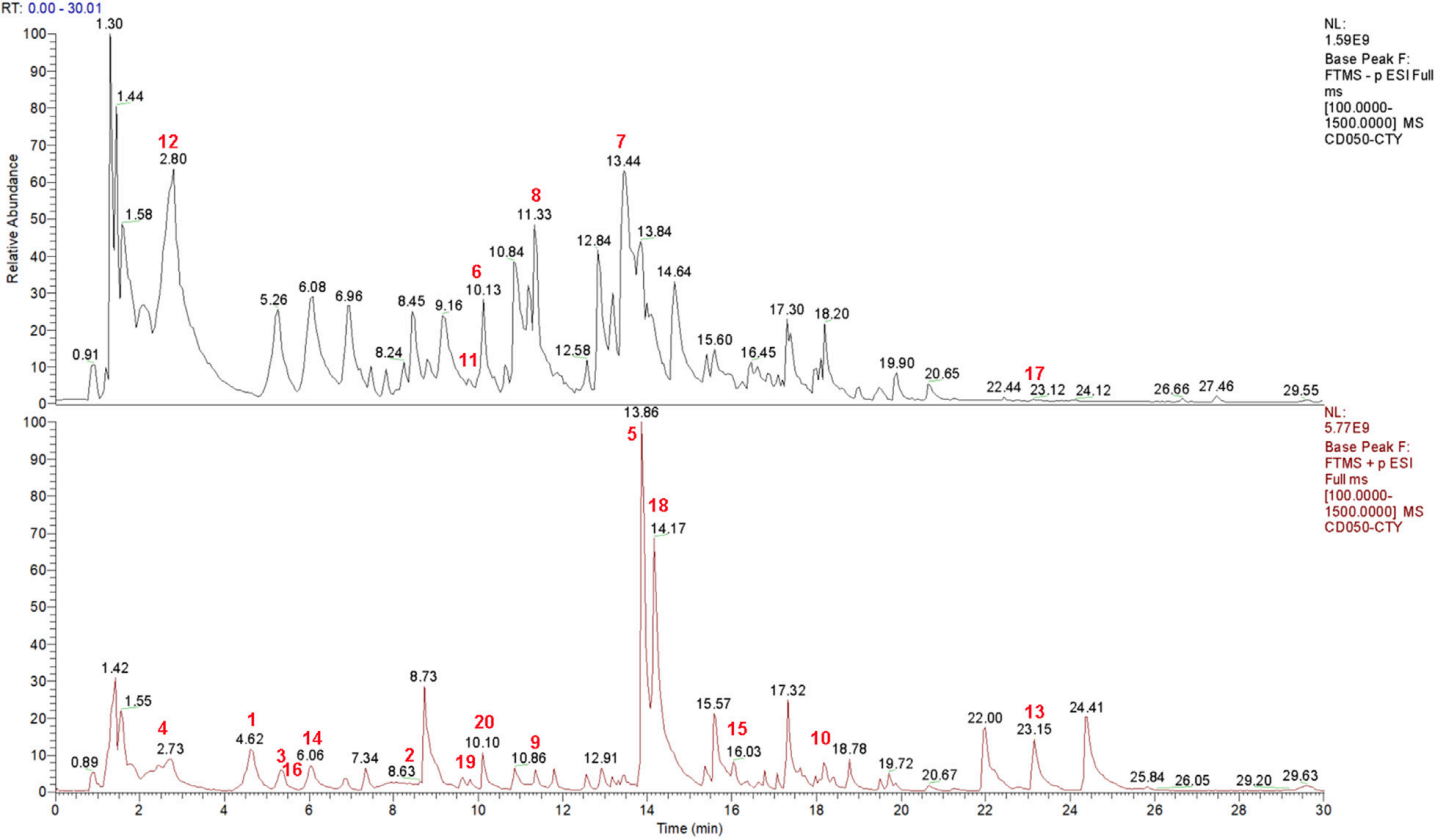
Chemical components of the Bushen Jianpi Huoxue Formula analyzed by HPLC-MS. Total ion current diagrams are presented for negative ion mode and positive ion mode. The top 20 compounds are highlighted with red numbers.

Qualification analysis was conducted using a multiple reaction monitoring (MRM) system, which includes two components: the selection of the precursor ion (MS 1) and the selection of a specific fragment of that precursor ion (MS 2). The data collected from the high-resolution liquid chromatography were initially compiled using CD2.1 and compared to the mzCloud database. Figure 1 showed the representative total ion current chromatograms obtained in positive and negative ion electrospray. Table 2 showed the top 20 compounds from the formula, which are more than 90% consistent with the compounds published in the mzCloud database.

**TABLE 2:** Primary compounds in the Bushen Jianpi Huoxue Formula detected by HPLC-MS.

No	Compounds	Formula	RT (min)
1	Adenosine	C_10_H_13_N_5_O_4_	4.639
2	2,3,4,9-Tetrahydro-1H-β-carboline-3-carboxylic acid	C_12_H_12_N_2_O_2_	8.644
3	L-Phenylalanine	C_9_H_11_NO_2_	5.351
4	Nicotinic acid	C_6_H_5_NO_2_	2.059
5	Ononin	C_22_H_22_O_9_	13.444
6	Benzoic acid	C_7_H_6_O_2_	9.82
7	Salicylic acid	C_7_H_6_O_3_	13.047
8	2,3-Dihydro-1-benzofuran-2-carboxylic acid	C_9_H_8_O_3_	11.54
9	Daidzin	C_21_H_20_O_9_	11.269
10	Dipropyleneglycol dibenzoate	C_20_H_22_O_5_	18.245
11	Isophthalic acid	C_8_H_6_O_4_	10.037
12	4-Oxoproline	C_5_H_7_NO_3_	2.736
13	Di(2-ethylhexyl) phthalate	C_24_H_38_O_4_	23.155
14	Citraconic acid	C_5_H_6_O_4_	5.89
15	Formononetin	C_16_H_12_O_4_	15.901
16	Guanine	C_5_H_5_N_5_O	5.378
17	Oleic acid	C_18_H_34_O_2_	22.978
18	Daidzein	C_15_H_10_O_4_	14.159
19	Chlorogenic acid	C_16_H_18_O_9_	9.791
20	[(3R,5R,6S,8S)-3-(β-D-Glucopyranosyloxy)-6-hydroxy-8-methyl-9,10-dioxatetracyclo[4.3.1.02,5 03,8]dec-2-yl]methyl benzoate	C_23_H_28_O_11_	10.124

### Effects of Beclin-1 overexpression on osteoblast differentiation and mineralization

**FIGURE 2:**
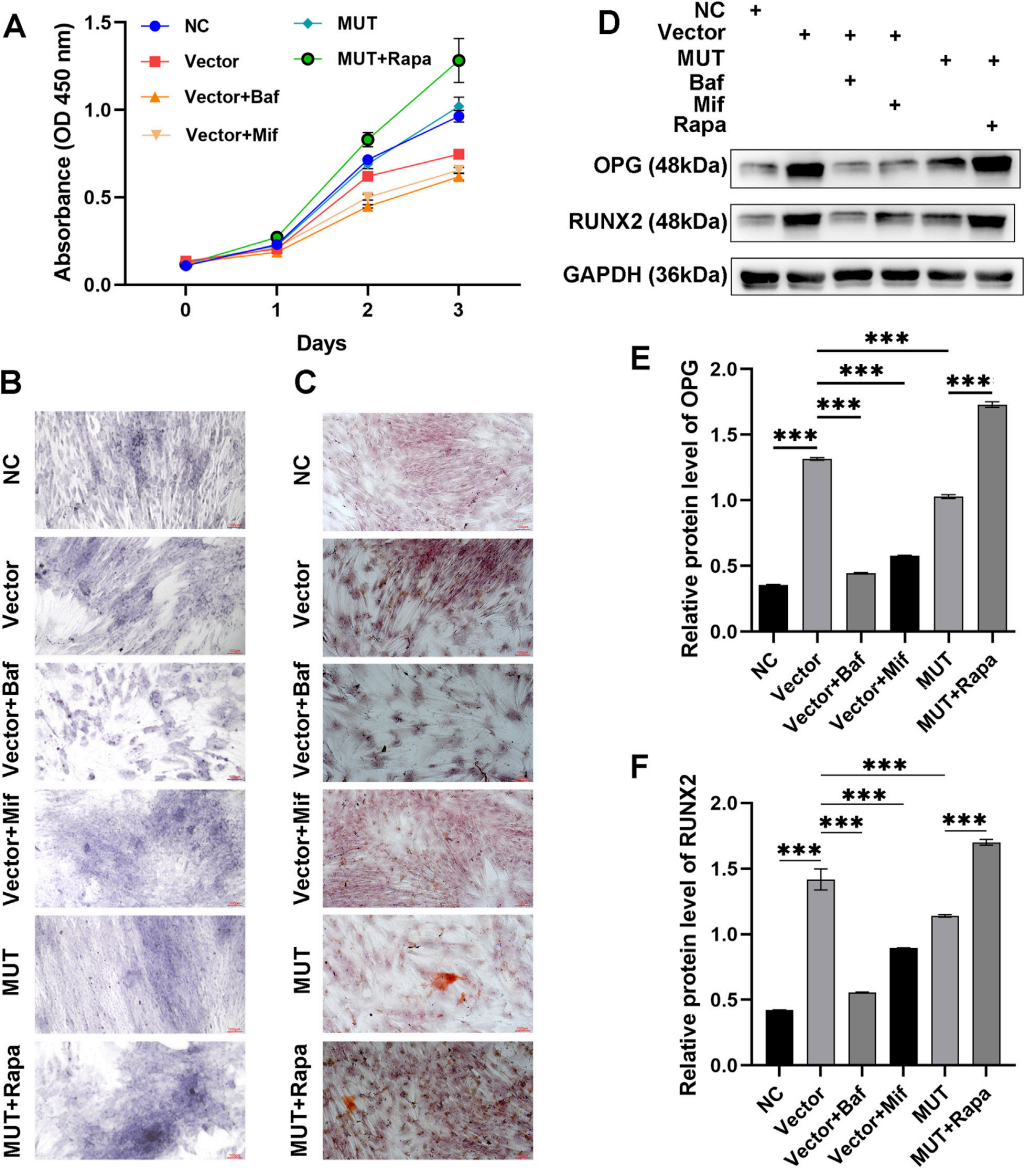
Beclin-1-mediated autophagy was associated with increased differentiation and mineralization of osteoblasts. **(A)** Cell viability of osteoblasts assayed by CCK-8 method. **(B)** Osteoblasts stained by alkaline phosphatase (ALP) staining to observe osteogenic differentiation. **(C)** Osteoblasts stained by Alizarin Red S (ARS) to observe bone mineralized nodules. **(D)** Immunoblotting assay to detect the expression of osteogenesis-related proteins (OPG and RUNX2), in the osteoblasts. Relative protein expression of OPG **(E)** and RUNX2 **(F)** in the indicated groups. MUT, osteoblasts cells with Beclin-1 overexpression; Bafilomycin A1 (Baf), autophagy inhibitor; Mifepristone (Mif), autophagy activator; Rapamycin (Rapa), autophagy activator. ****p < 0.0001.

To investigate the effects of Beclin-1/Bcl-2 binding on autophagy and apoptosis of osteoblasts, this study utilized Baf to inhibit autophagy, and used Mif to block the binding of Beclin-1 to Bcl-2. Compared to the vector cells, Baf and Mif did not decreased the cell viability of osteoblasts (p > 0.05) (Figure 2A). These results ensured that the effects of the Beclin-1/Bcl-2 complex on autophagy and apoptosis are not affected by cell viability. The CCK-8 assay indicated that by day 3, Beclin-1 overexpression significantly increased cell activity of osteoblasts, compared to the vector cells (p > 0.05). Rapa is an autophagy activator that was utilized in this study to stimulate autophagy. Compared to the MUT cells, Rapa did not significantly increase the cell viability of MUT osteoblasts (p > 0.05), which excluded the effects of Rapa on cell viability of osteoblasts.

ALP staining was performed to assess the osteogenic differentiation capacity. ALP staining showed that compared to the vector cells, MUT cells demonstrated increased ability to differentiate (Figure 2B). Notably, the differentiation of MUT osteoblasts was further enhanced after Rapa (autophagy activator) treatment. Administration of Baf (autophagy inhibitor) inhibited the differentiation of osteoblasts. Compared to the Baf, administration with Mif (autophagy activator) promoted the differentiation of osteoblasts. These results indicated that Beclin-1 overexpression may increase the differentiation of osteoblasts through interfering autophagy process. ARS staining was carried out to assess the ability of osteoblasts to form a mineralizing matrix. Figure 2C showed that compared to the vector group, Beclin-1 overexpression promoted osteogenic mineralization. Treatment with Rapa further increased the mineralization in MUT osteoblasts. Hence, osteogenic mineralization induced by Beclin-1 overexpression may be related to autophagy.

To assess the effects of Beclin-1 overexpression on osteogenesis-related genes, we analyzed the relative protein expression of osteogenic genes (*OPG* and *RUNX2*) using immunoblotting method (Figures 2D–F). Osteoblasts secrete a soluble decoy receptor, OPG, that blocks RANK/RANKL interaction by binding to RANKL and, thus, prevents osteoclast differentiation and activation. Normally, RUNX2 in osteoblasts reduces during bone development, which are required for mature bone formation. RUNX2 triggers the expression of major bone matrix genes during the early stage of osteoblast differentiation. Compared to the vector group, there was a significant decrease in the protein expression of *OPG* and *RUNX2* in the MUT cells. However, osteoblasts in the MUT+Rapa group exhibited increased protein level of OPG and RUNX2. This demonstrated that enhanced autophagy in osteoblasts could improve the osteogenic differentiation and mineralization ability by up-regulating expression of OPG and RUNX2.

### Effects of the Bushen Jianpi Huoxue Formula on autophagy of osteoblasts

**FIGURE 3:**
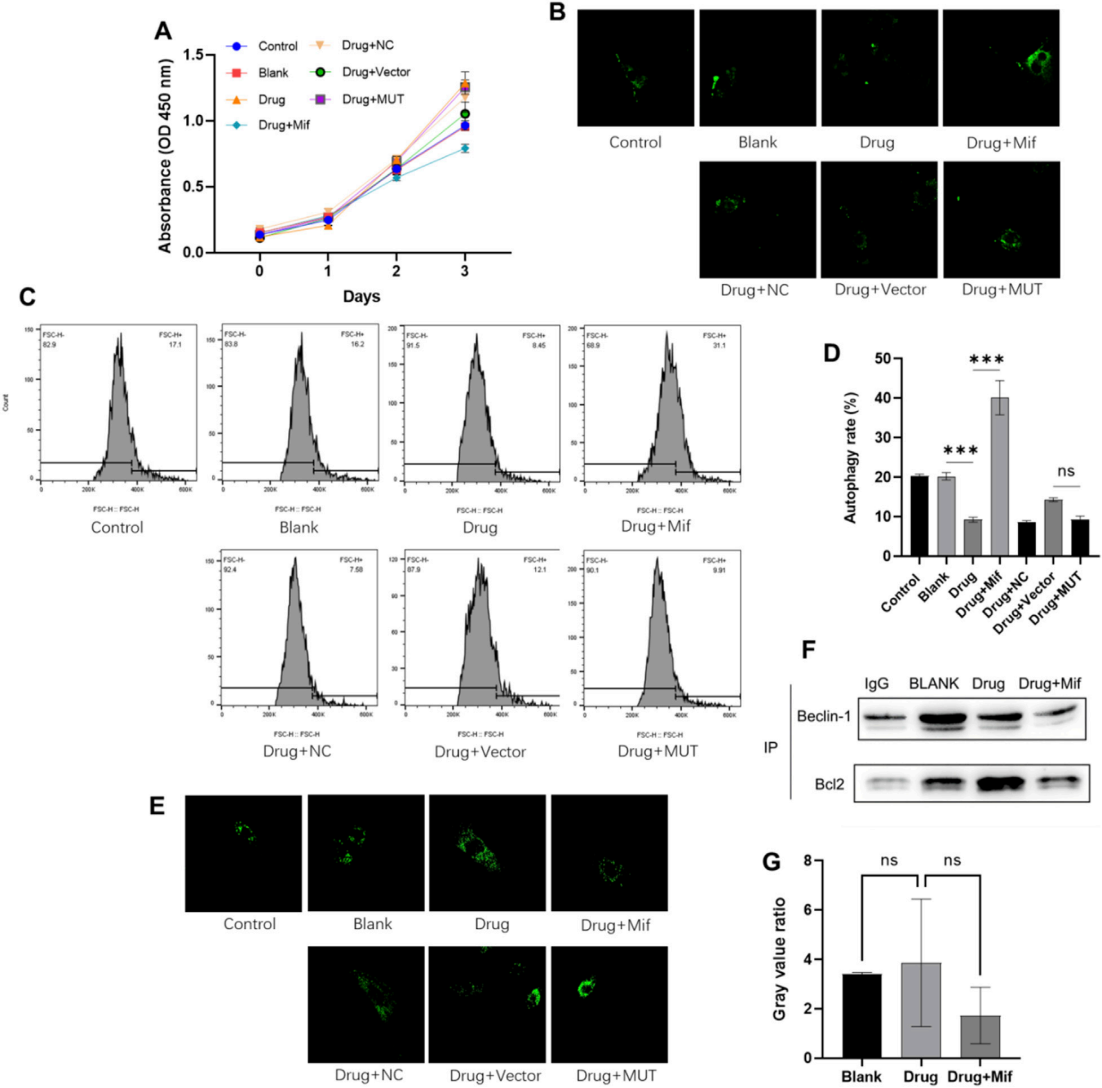
The Bushen Jianpi Huoxue Formula inhibited cellular autophagy while was not through promoting the binding of Beclin-1 and Bcl-2. **(A)** CCK-8 assay detected the cell viability of osteoblasts; **(B)** Autophagic vacuoles labelled with MDC; **(C)** MDC-labelled cells detected by flow cytometry; **(D)** Quantification of autophagy rate; **(E)** Lysosomes stained by LysoTracker probe; **(F)** The binding of Beclin-1 to Bcl-2 detected by immunoprecipitation. **(G)** Quantification of Beclin-1 and Bcl-2 complex. MUT, osteoblasts cells with Beclin-1 overexpression; Mifepristone (Mif), autophagy activator. ^ns^p > 0.05; ****p < 0.0001.

Firstly, CCK-8 assay was carried out to study the effects of formula on cell viability of osteoblasts. Figure 3A showed that compared to the Blank cells, osteoblasts treated with Drug exhibited increased cell viability on day 3 (p < 0.0001). Application of Mif decreased cell viability compared to the Drug group (p < 0.0001), which indicated that the drug may elevate cell viability through affecting the binding of Beclin-1 and Bcl-2. Besides, compared to the vector group, cell viability of MUT cells was increased after treatment with drug (p < 0.0001). This result suggested that the effects of drug on cell viability were associated with Beclin-1 expression.

To investigate the impact of herbal intervention on macroautophagy in osteoblasts, autophagic vesicles were stained using MDC staining and observed by flow cytometry. Compared to the Blank cells, drug treatment can reduce the autophagy rate by 50% (p < 0.0001) (Figures 3B–D). Compared to the Drug group, the combined application of Mif increased autophagy rate of osteoblast (p < 0.0001). However, application of the formula showed no effects on autophagy rate of MUT cells compared to osteoblasts in the Drug + Vector group (p > 0.0001). Consistently, lysosomal staining indicated no significant differences between the Drug + Vector and Drug + MUT groups (Figure 3E). Then, immunoprecipitation was employed to assess the levels of the Beclin-1/Bcl-2 complex in osteoblasts with or without drug treatment. The findings indicated that treatment with drug did not increase the binding of Beclin-1 to Bcl-2 (Figures 3F, G). These results confirmed that the Bushen Jianpi Huoxue Formula inhibiting the rate of cellular autophagy, autophagic vesicle formation, or lysosomal biogenesis, while was not through affecting binding of Beclin-1 and Bcl-2.

### Effects of the Bushen Jianpi Huoxue Formula on osteoblast apoptosis

**FIGURE 4:**
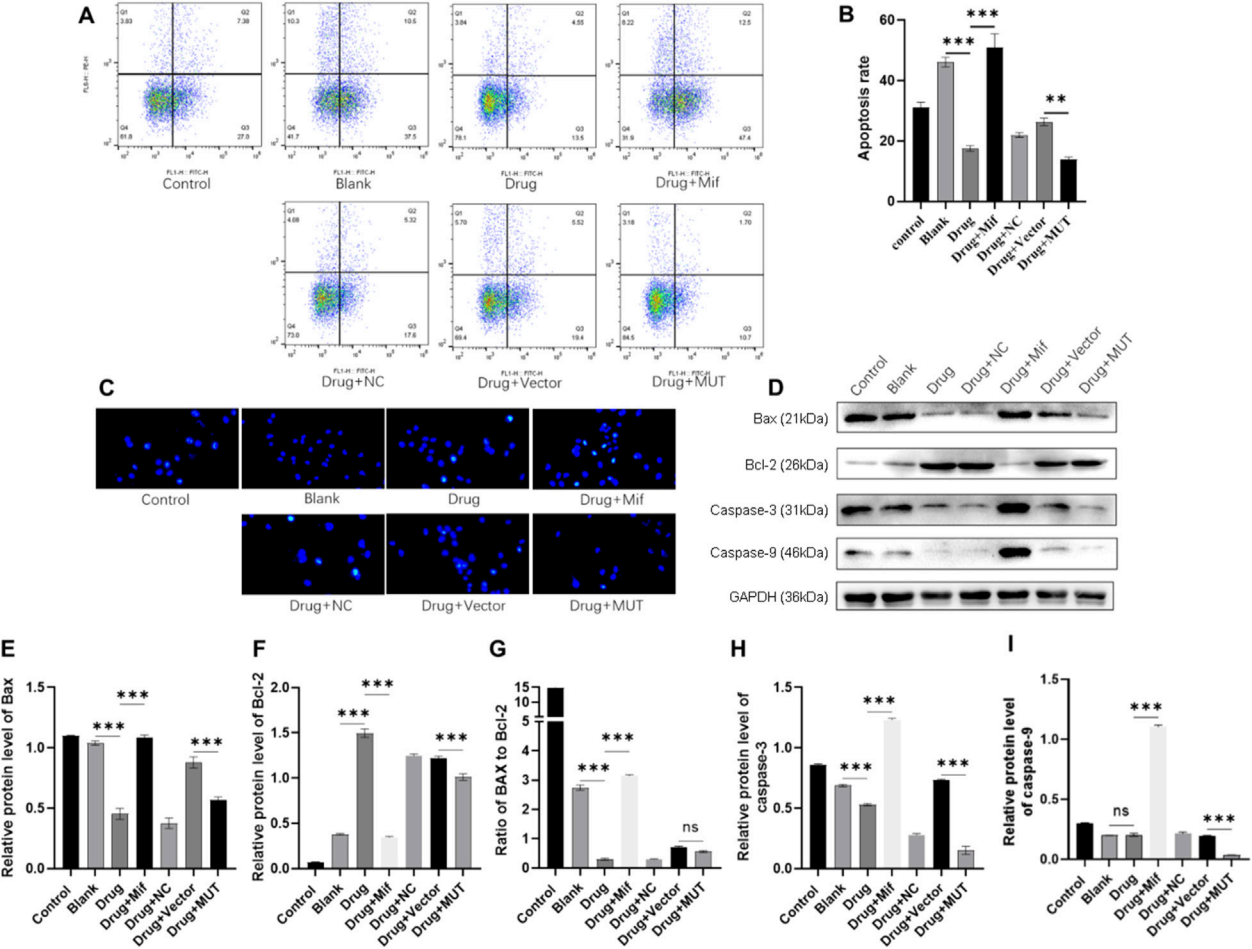
Effects of the Bushen Jianpi Huoxue Formula on apoptosis of osteoblasts. **(A)** Apoptotic cells were marked by Annexin V-PE/7-AAD and observed by flow cytometry. **(B)** Quantitative analysis of apoptosis rate of osteoblasts detected by flow cytometry. **(C)** Hoechest staining was performed to observe apoptotic cells. **(D)** Immunoblotting assay to detect the expression of apoptotic proteins (Bax, Bcl-2, Bax/Bcl-2, Caspase-3, and Caspase-9) in osteoblasts. Relative expression of Bax **(E)**, Bcl-2 **(F)**, Bax **(G)**, Caspase-3 **(H)**, and Caspase-9 **(I)** was quantitatively presented based on immunoblotting assay. MUT, osteoblasts cells with Beclin-1 overexpression; Mifepristone (Mif), autophagy activator. ^ns^p > 0.05, **p < 0.01, ****p < 0.0001.

To assess the effects of the Bushen Jianpi Huoxue Formula intervention on apoptosis of osteoblasts, flow cytometry analysis was carried out to analyze apoptosis rate. Compared to the Blank cells, drug treatment significantly inhibited apoptosis of osteoblasts (p < 0.0001) (Figures 4A, B). However, WT osteoblasts were more sensitive to the drug treatment in comparison to MUT osteoblasts (p < 0.01). This finding suggested that the Bushen Jianpi Huoxue Formula intervention can enhance the inhibition of apoptosis induced by Beclin-1 overexpression. We observed the morphological changes of apoptosis in each group by immunofluorescence staining. As shown in Figure 4C, following the intervention of Bushen Jianpi Huoxue Formula, the apoptotic rate of MUT cells was significantly decreased.

To assess the expression of apoptotic proteins, the relative expression of Bax, Bcl-2, caspase-3, and caspase-9, and ratio of Bax to Bcl-2 were analyzed. Compared to the Blank group, drug treatment significantly decreased the protein expression of Bax and caspase-3, increased the protein expression of Bcl-2, and decreased the ratio of Bax to Bcl-2 (Figures 4D–I). In contrast, Mif treatment inhibited the effects of the drug on the protein level of apoptotic proteins. WT and MUT cells were also treated with the drug to confirm whether the drug plays roles in apoptosis via Beclin-1. Compared to the Vector group, protein expression of Bax, Bcl-2, caspase-3, and caspase-9 was decreased in the MUT cells after drug treatment. In summary, the Bushen Jianpi Huoxue formula can inhibit osteoblast apoptosis, and the overexpression of the Beclin-1 gene can enhance this function.

### Effects of the intervention of the formula for Bushen Jianpi Huoxue on the differentiation and mineralization of osteoblasts via Beclin-1

**FIGURE 5:**
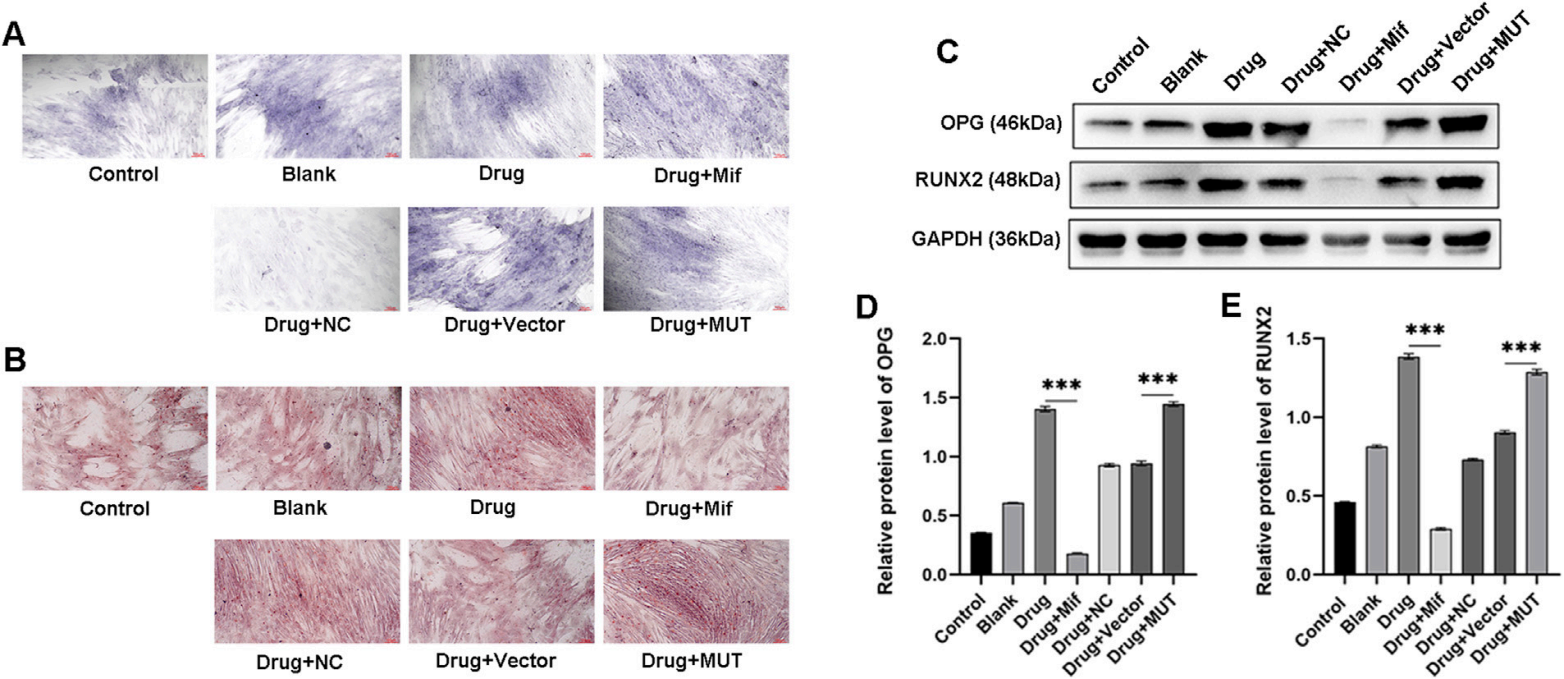
Effects of the intervention of the formula of Bushen Jianpi Huoxue Formula on the differentiation and mineralization of osteoblasts. **(A)** ALP staining was used to observe the mineralized nodules of osteoblasts. **(B)** Alizarin red staining was used to observe the mineralized nodules of bones. **(C)** Osteogenesis-related proteins (OPG and RUNX2) in osteoblasts detected by immunoblotting assay. Relative expression of OPG **(D)** and RUNX2 **(E)** in osteoblasts of the indicated groups. MUT, osteoblasts cells with Beclin-1 overexpression; Mifepristone (Mif), autophagy activator. ****p < 0.0001.

To investigate the effects of Bushen Jianpi Huoxue Formula on osteoblast differentiation and mineralization, ALP staining and ARS staining were performed. Results of ALP staining revealed enhanced differentiation of osteoblasts after treatment with the formula, compared to the Blank group (Figure 5A). In the MUT cells, application of the drug further promoted differentiation of osteoblasts compared to the vector group. ARS staining showed enhanced osteogenic mineralization in the drug group, compared with the blank group (Figure 5B). In contrast, the Drug+Mif group exhibited significantly reduced mineralization capacity. Bushen Jianpi Huoxue Formula significantly improved osteogenic mineralization in the MUT group compared to the vector group. This enhancement was accompanied by elevated expression levels of osteogenesis-related genes (*OPG* and *RUNX2*) (Figures 5C–E). This comprehensive approach highlights the formula’s potential in enhancing osteoblast activity and supports its therapeutic application in bone regeneration and osteoporosis treatment.

### Effects of the Bushen Jianpi Huoxue Formula intervention on endoplasmic reticulum stress via Beclin-1

**FIGURE 6:**
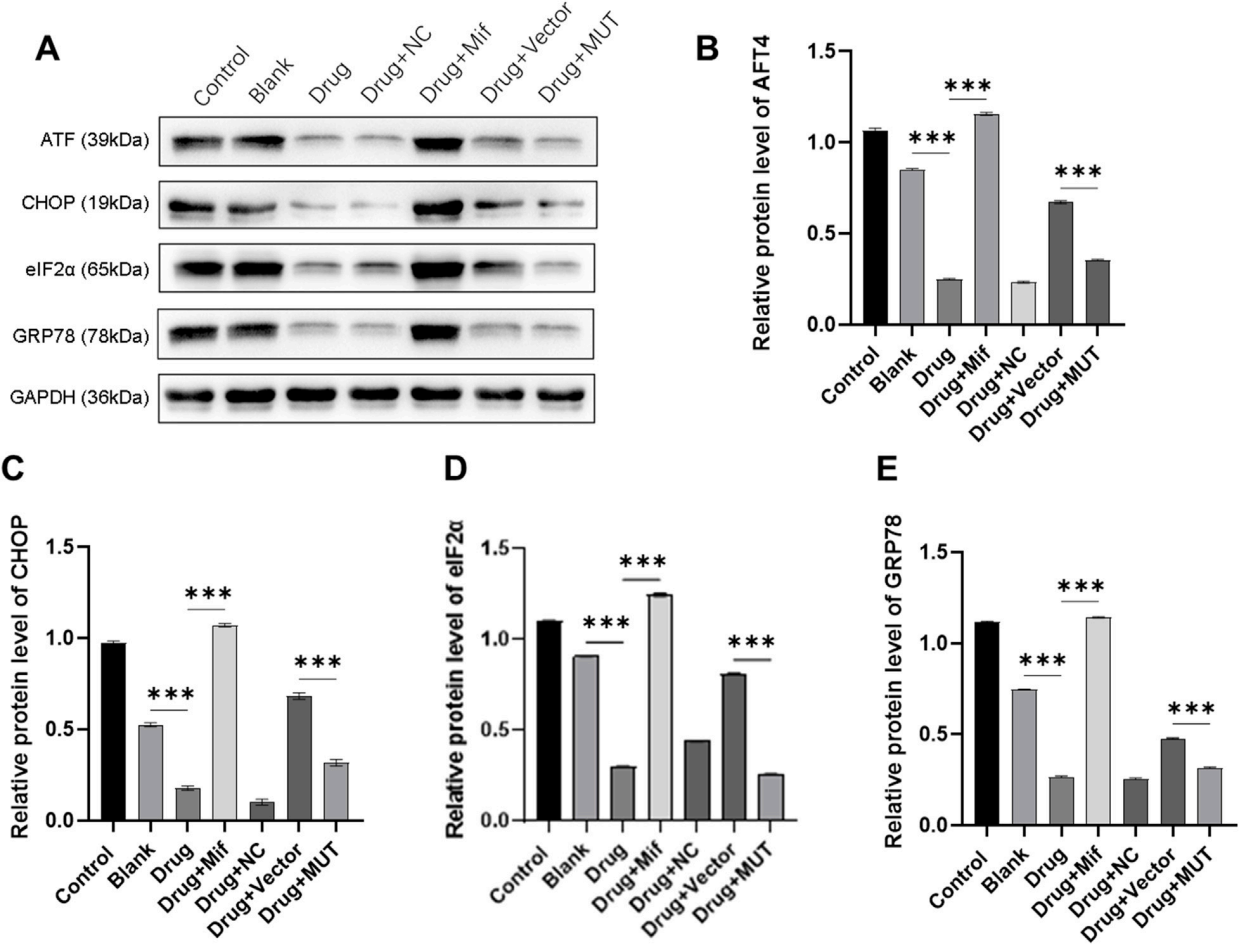
Effects of the intervention of the formula of Bushen Jianpi Huoxue Formula on the protein expression of endoplasmic reticulum stress-related gene. **(A)** Immunoblotting assay for detecting protein expression of osteogenesis-related genes in osteoblasts; Relative protein expression of AFT4 **(B)**, CHOP **(C)**, eIF2α **(D)**, GRP78 **(E)** were quantified according the results from immunoblotting assay. MUT, osteoblasts cells with Beclin-1 overexpression; Mifepristone (Mif), autophagy activator. ****p < 0.0001.

To further understand the potential function mechanism of the Bushen Jianpi Huoxue formula, we detected the expression levels of key proteins (ATF4, CHOP, eIF2α, and GRP78) in PERK pathway involved in the regulation of endoplasmic reticulum stress. Western blot results showed that the formula significantly decreased the protein expression of ATF4, CHOP, eIF2α, and GRP78 compared to the Blank group (p < 0.0001) (Figures 6A–E). However, treatment with Mif reversely increased the protein expression in the Drug + Mif group compared to the Drug group (p < 0.0001). In the MUT group, the protein expression of ATF4, CHOP, eIF2α, and GRP78 was significantly reduced by the formula compared to the Vector group (p < 0.0001). This finding suggested that the formula can inhibit endoplasmic reticulum stress through the PERK pathway.

### Effects of the Bushen Jianpi Huoxue Formula intervention on osteogenesis-related protein via Beclin-1

**FIGURE 7:**
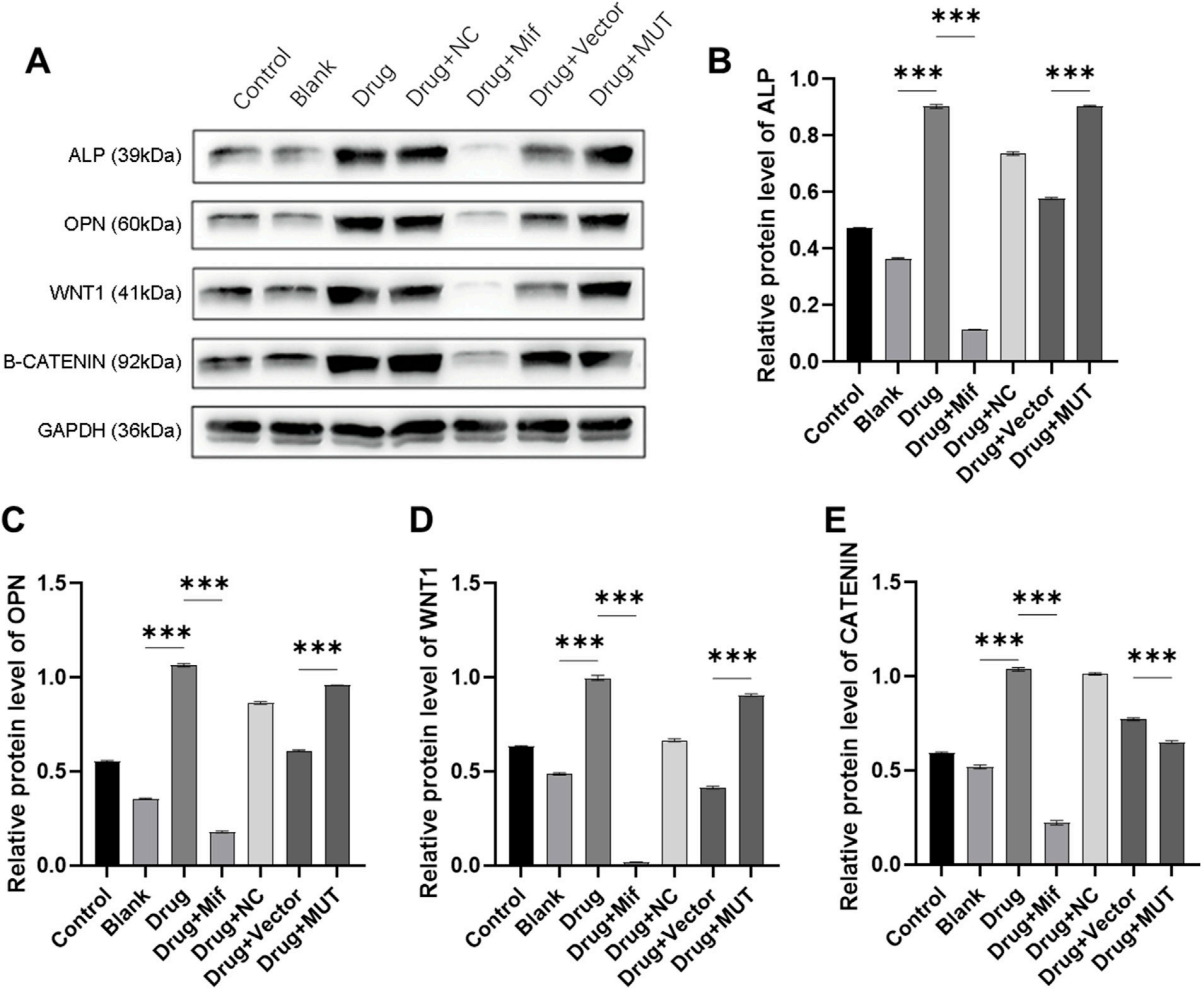
Effects of the intervention of the formula of Bushen Jianpi Huoxue Formula on WNT/β-CATENIN pathway. **(A)** Immunoblotting assay for detecting protein expression of osteogenesis-related genes in osteoblasts; Relative protein expression of ALP **(B)**, OPN **(C)**, WNT1 **(D)**, β-CATENIN **(E)** were quantified according the results from immunoblotting assay. MUT, osteoblasts cells with Beclin-1 overexpression; Mifepristone (Mif), autophagy activator. p < 0.0001.

To study the effects of the formula on osteogenesis, protein expression of ALP, OPN, WNT1, and β-CATENIN was assayed. As shown in Figures 7A–E, in drug-treated osteoblasts, protein expression of osteogenesis-related genes was significantly increased (p < 0.0001). It was found that after treatment with Mif, effects of the formula on protein expression were inhibited (p < 0.0001). For the MUT cells, the formula significantly decreased the protein expression of osteogenesis-related protein, compared to the Drug + Vector group (p < 0.0001).

## Discussion

Osteoporosis is a systematic bone disability characterized by low bone density and microarchitectural deterioration of boney tissue. The current available pharmacological treatments for osteoporosis are anti-resorptive (inhibiting the osteoclasts), bone forming (stimulating the osteoblasts) or dual acting (simultaneously stimulating the osteoblasts and inhibiting the osteoclasts). However, there still are challenges in promoting osteoblastogenesis and bone formation. Bone formation is dependent on the recruitment of sufficient number of osteoblasts as well as the activity of individual osteoblasts. The anabolic therapies can increase bone formation by increasing the number or activity of mature osteoblasts or by preventing their apoptosis. Nevertheless, only few of them appear to be suitable and safe for long-term systemic therapy due to low specificity, non-skeletal effects, potential side effects, and cost.

### The role of Beclin-1/Bcl-2 complex

Bcl-2 serves as a negative regulator of Beclin-1 by directly binding to Beclin-1 and inhibiting its activity. Bcl-2 binds to the BH3 domain of Beclin-1, and thereby regulates autophagy in the endoplasmic reticulum. This interaction prevents Beclin-1 from promoting autophagy, thereby influencing cellular survival and death pathways. Dissociation of this complex has been demonstrated to enhance autophagy and inhibit apoptosis (Zhou et al., 2020). The interaction between Beclin-1 and Bcl-2 has been shown to be influenced by proteins such as Klotho, which in turn affects autophagy activity and helps cells resist oxidative stress (Li et al., 2020). In gliomas, the formation of the Beclin-1/Bcl-2 complex can be targeted using PI3K and Raf inhibitors to shift the balance between apoptosis and autophagy (Zając et al., 2023). However, there is limited research on the role of the Beclin-1/Bcl-2 complex in osteoblasts in the context of osteoporosis. In this study, we have successfully constructed an osteoblast model with Beclin-1 overexpression. After induction of autophagy with Rap, we observed a positive correlation between Beclin-1 overexpression and cell viability of osteoblasts, differentiation, and mineralization. Besides, Beclin-1/Bcl-2 complex may affect the differentiation and mineralization of osteoblasts.

### The effects of BJHF on osteoblasts

The TCM has already been utilized for the management of osteoporosis, possessing equally anabolic and anticatabolic impacts by boosting osteogenesis and minimizing extremely unbalanced bone turnover (Zhang N-D. et al., 2016). According to the zàng-fu theory of the TCM, the kidney’s primary duties are to build bones, control human growth and development, and generate marrow to fill the brain (Li and Xu, 2011). Numerous kidney-nourishing herbal medications can restore bones, as per the Chinese medicine kidney theory, and are thus prescribed to cure bone-related disorders including osteoporosis (He et al., 2017; Duan et al., 2023). Besides, published research found that TCM for the therapy of osteoporosis seem to minimize unbalance osteoclast activity, enhance bone mineral density and biomechanical properties, and diminish bone microstructural degeneration (Zhang et al., 2008; Lin et al., 2002).

Herbal medication components of the Bushen Jianpi Huoxue Formula in this study contain Fructus Psoraleae, Epimedii Folium, Desertliving Cistanche, Prepared Rehmannia Radix, Radix paeoniae alba, Astragali Radix, Semen Cuscuta, Radix Salviae miltiorrhizae, Angelica sinensis, and Jujube. It has been proved that these ingredients can effectively improve the symptoms of osteoporosis (Ou et al., 2021; Yang et al., 2022; Liang et al., 2017; Zhang et al., 2019; Lin et al., 2024; Sun et al., 2024; Kim et al., 2014; Yang et al., 2002; Hwang et al., 2021; Algandaby, 2019). The molecular mechanism may be closely associated with the estrogen signaling pathway (Ou et al., 2021), inhibition of bone resorption by osteoclasts (Yang et al., 2022), induction of osteoblast proliferation (Liang et al., 2017), induction of osteoblast proliferation, differentiation, and mineralization (Zhang et al., 2019; Lin et al., 2024; Sun et al., 2024; Kim et al., 2014; Yang et al., 2002), inhibition of osteoclastogenesis (Hwang et al., 2021), and attenuation of metabolic syndrome (Algandaby, 2019). From the perspective of TCM, the chemical constituents of these herbal medicines replenish the kidney, strengthen the spleen, activate blood circulation and dissipate blood stasis, which explains the mechanism of their anti-osteoporotic activity (Zhang ND. et al., 2016). From the cellular perspective, the formula is effective in inhibiting autophagy and apoptosis, while significantly increases osteoblastic differentiation and mineralization, which further illustrated the anti-osteoporotic mechanism.

### The involvement of the WNT/β-CATENIN

The WNT/β-CATENIN is a classical signaling pathway that regulates stem cell differentiation and is a principal pathway utilized in TCM to address osteoporosis. This underscores its importance in modulating cellular processes of bone health and highlights the potential of TCM in targeting specific molecular pathways to treat osteoporosis. Our findings demonstrated that the Bushen Jianpi Huoxue Formula could enhance the differentiation and mineralization ability of osteoblasts by up-regulating the expression of the WNT/β-CATENIN pathway, which is mediated by Beclin-1 overexpression. Furthermore, the formula may activate the WNT/β-CATENIN pathway by inhibiting autophagy, which could be linked to the promotion of the formation of the Beclin-1 and Bcl-2 complex.

### The mitigation of ER stress

The endoplasmic reticulum (ER) is the site of nutrient synthesis and an important pathway for protein folding and modification. ER stress occurs when there is misfolding of ER luminal proteins, aggregation of unfolded proteins, and disruption of calcium-ion homeostasis (Zhong et al., 2023). ER stress impacts bone metabolism, and PERK plays a pivotal role in this process. Dissociation of GRP78 from PERK facilitates its oligomerization and activation, thereby triggering downstream effectors such as eIF2α, CHOP, and ATF4, which are crucial for osteoblast development (Guo et al., 2021). Further research has demonstrated that early autophagy mitigates oxidative damage in osteoblasts via the ER stress pathway (Yang et al., 2014). Alterations in calcium homeostasis and the accumulation of misfolded proteins in the endoplasmic reticulum induce ER stress, potentially leading to activation of caspase-12 and/or Bcl-2-associated death promoter, culminating in apoptosis (Huang et al., 2023). In this study, we noted a marked reduction in PERK pathway protein levels in MUT cells compared to their WT counterparts within the same group, implying that Beclin-1 overexpression in osteoblasts may diminish the accumulation of intracellular misfolded proteins, thereby alleviating ER stress (Fernández et al., 2015).

## Conclusion

In conclusion, the Bushen Jianpi Huoxue formula significantly enhanced the proliferative activity and osteoblastic differentiation and mineralization induced by Beclin-1 overexpression, which may be through the activation of the WNT/β-CATENIN pathway. The present study experimentally demonstrated the effect of Beclin-1/Bcl-2 on osteoblasts, which provided new ideas and theoretical basis for understanding the pathogenesis of osteoporosis. Furthermore, the formula reduced the apoptotic death of osteoblasts, inhibited the PERK signaling pathway, and downregulated ER stress, which provides new ideas for the prevention and treatment of osteoporosis. However, this study has several limitations, including the use of a single cell line, the potential for confounding variables, and the necessity for further validation *in vivo*.

## Data Availability

The datasets used and/or analysed during the current study are available from the corresponding author on reasonable request.

## Abbreviations

BH, Bcl-2 Homology; TCM, Traditional Chinese Medicine; BMD, Bone Mineral Density; ALP, Alkaline phosphatase; ARS, Alizarin Red S; HPLC-MS, High performance liquid chromatography-mass spectrometry; MUT, mutant; Rapa, Rapamycin; Baf, Bafilomycin A1; Mif, Mifepristone; CCK-8, Cell counting kit-8; MDC, Monodansylcadaverine; ER, endoplasmic reticulum.
